# Clinical Manifestations and Epigenetic Regulation of Oral Herpesvirus Infections

**DOI:** 10.3390/v13040681

**Published:** 2021-04-15

**Authors:** Natalie Atyeo, Michelle D. Rodriguez, Bernadett Papp, Zsolt Toth

**Affiliations:** 1 Department of Oral Biology, University of Florida College of Dentistry, 1395 Center Drive, Gainesville, FL 32610, USA; natyeo@dental.ufl.edu (N.A.); MRodriguez@dental.ufl.edu (M.D.R.); 2 Genetics Institute, University of Florida, Gainesville, FL 32610, USA; 3 Health Cancer Center, University of Florida, Gainesville, FL 32610, USA; 4 Informatics Institute, University of Florida, Gainesville, FL 32610, USA; 5 Center for Orphaned Autoimmune Disorders, University of Florida, Gainesville, FL 32610, USA

**Keywords:** herpesviruses, oral infection, HSV-1, KSHV, EBV, epigenetic viral gene regulation, viral chromatin, polycomb group proteins

## Abstract

The oral cavity is often the first site where viruses interact with the human body. The oral epithelium is a major site of viral entry, replication and spread to other cell types, where chronic infection can be established. In addition, saliva has been shown as a primary route of person-to-person transmission for many viruses. From a clinical perspective, viral infection can lead to several oral manifestations, ranging from common intraoral lesions to tumors. Despite the clinical and biological relevance of initial oral infection, little is known about the mechanism of regulation of the viral life cycle in the oral cavity. Several viruses utilize host epigenetic machinery to promote their own life cycle. Importantly, viral hijacking of host chromatin-modifying enzymes can also lead to the dysregulation of host factors and in the case of oncogenic viruses may ultimately play a role in promoting tumorigenesis. Given the known roles of epigenetic regulation of viral infection, epigenetic-targeted antiviral therapy has been recently explored as a therapeutic option for chronic viral infection. In this review, we highlight three herpesviruses with known roles in oral infection, including herpes simplex virus type 1, Epstein–Barr virus and Kaposi’s sarcoma-associated herpesvirus. We focus on the respective oral clinical manifestations of these viruses and their epigenetic regulation, with a specific emphasis on the viral life cycle in the oral epithelium.

## 1. Introduction

**Figure 1:**
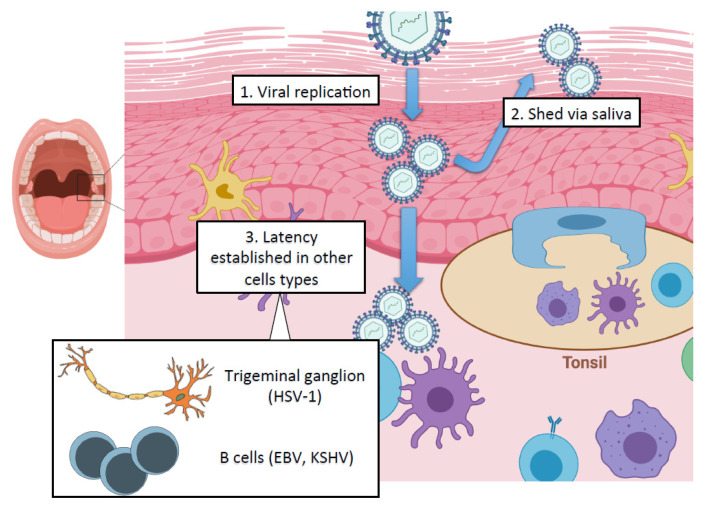
The oral epithelium and virus challenge: the oral epithelium is often an initial site for viral entry and provides a site for viral replication (1) and subsequent shedding via saliva (2) or spread to other cell types in the underlying connective tissue (3), wherein some viruses, such as the herpesvirus family, can establish chronic infections. Inset: Herpes simplex virus-1 (HSV-1) establishes lifelong latent infection in neurons of the trigeminal ganglion; gammaherpesviruses Epstein–Barr virus (EBV) and Kaposi’s sarcoma-associated herpesvirus (KSHV) establish latent infection in B cells. The figure was created with BioRender.

The oral cavity is the initial site of contact for many viruses, permitting viral replication in the oral epithelium and subsequent spread to other cell types in the adjacent soft tissue (Figure 1). While the oral stratified squamous epithelium and deeper connective tissue layers serve as potent mechanical barriers against infection, breaches in this barrier can allow access to cellular receptors for viral fusion and entry [1]. The pathologic inflammation initiated by periodontal pathogens in the oral cavity also facilitates the breakdown of tight epithelial barriers, which may promote viral entry [2]. In addition to their roles as mechanical barriers, cells of the oral mucosa release cytokines, chemokines and antimicrobial peptides, which protect against viral challenge [3,4]. Along with the cells of the oral mucosa, saliva forms an essential component of the innate immune system, and antibacterial and antiviral factors are readily detected in saliva [5].

Advances in viral detection through highly sensitive PCR technologies have led to the identification of many different viruses in the saliva, gingival crevicular fluid and throat wash samples, indicating that infectious viruses may be present in the oral cavity at a greater rate than previously recognized [6]. The presence of infectious viruses in the oral cavity is of epidemiologic significance, as resulting viral shed via saliva is often the major route of virus transmission between individuals, such as in the case of herpesviruses [7,8,9]. Clinically, increasing evidence supports a key role for viral pathogens in the development of oral pathologies, including intraoral lesions, periodontal disease and oral tumor development [10,11]. These oral diseases are usually the outcome of virus-induced direct cytotoxic effect or damage from the host’s antiviral immune response [12]. Additionally, systemic immunosuppression, as in the case of HIV infection, can elicit the development of secondary oral manifestations, including reactivation of secondary oral viral infections [13]. The oral effects of HIV infection are numerous and will only be discussed in this review in the context of co-infection with other oral viruses, as the oral clinical manifestations of HIV have been reviewed extensively [14,15]. Despite the biological and clinical relevance of oral viral infection, mechanistic studies on viral infections of the oral cavity are lacking.

An emerging field of research has focused on the importance of epigenetic regulation of the viral life cycle and on the influence of epigenetics on the outcomes of viral infections [16]. Importantly, virus-mediated dysregulation of host epigenetic control may impact a wide variety of cell functions, including the cell cycle, DNA damage response pathway and immune response. As a result, epigenetic-targeted drug therapies are promising options for the treatment of chronic viral infections [17]. While the field of viral epigenetics has grown in recent years, there is still limited understanding of the role of epigenetics in viral infections of the oral cavity. This review will examine relevant oral viral pathogens, with an emphasis on the human herpesvirus family, and the subsequent implications in epigenetics and drug development.

## 2. Oral Manifestations of Herpesvirus Infections

Some of the most well-characterized viral pathogens with respect to oral manifestations are the members of the herpesvirus family, which includes eight viruses with known human pathogenicity: herpes simplex virus type 1 and herpes simplex virus type 2 (HSV-1, HSV-2), varicella-zoster virus (VZV), human cytomegalovirus (HCMV), Epstein–Barr virus (EBV), human herpesvirus-6 and human herpesvirus-7 (HHV-6, HHV-7) and Kaposi’s sarcoma-associated herpesvirus (KSHV). Herpesviruses are large double-stranded DNA viruses that are highly prevalent pathogens, causing lifelong persistent infection. After initial lytic infection, herpesviruses establish latency in specific cell types of the infected host. The importance of latency is that it allows herpesvirus evasion of the host immune defenses and permits lifelong infection [18]. Importantly, herpesviruses can intermittently undergo lytic reactivation in latently infected cells upon immunosuppression or various external stimuli and stress factors resulting in virus replication and transmission [19]. Some studies have also implicated dental procedures as potential sources of increased shedding of HSV-1 into saliva and sometimes in extensive recurrence of herpes labialis [20,21]. However, it is still unclear what specific dental or anesthetic procedures trigger the increase in HSV-1 replication in the oral cavity.

Herpesviruses can be detected in oropharyngeal swabs, saliva samples, and have been isolated from gingival crevicular fluid samples from patients with periodontitis, indicating both a reservoir for herpesviruses in the oral cavity and a route for viral transmission between individuals [22,23,24]. Recently, herpesviruses have been implicated in the progression of periodontal disease in concert with bacterial pathogens, prompting a potential paradigm shift in periodontitis pathogenesis, which has been reviewed extensively elsewhere [25,26,27]. All human herpesviruses have been implicated in oral disease to an extent, but the prevalence of these oral clinical manifestations varies among virus families. Here, we focus on the three herpesviruses which are most commonly connected to the oral cavity in terms of cellular tropism, route of viral transmission and ability to produce significant oral pathologies. Information about the specific roles for other viruses in oral disease has been outlined in other reviews [11,28].

### 2.1. Herpes Simplex Virus Type 1 (HSV-1)

Primary infection with HSV-1 generally occurs during childhood, in which inoculation occurs through salivary spread or contact with an active lesion [29]. Initial exposure to HSV-1 may be asymptomatic or present clinically as herpetic gingivostomatitis, with possible concurrent fever, lymphadenopathy and other constitutional signs and symptoms [30]. Primary infection with HSV-1 is commonly coupled with the appearance of oral lesions, which can affect both keratinized and non-keratinized tissues in the oral cavity and are often localized on the gingiva, buccal mucosa and hard and soft palate [31]. After primary infection, which typically resolves within a week, the virus maintains latency in sensory neurons of the trigeminal ganglion, in the case of orofacial HSV-1 infection [32]. HSV-1 can be subsequently reactivated from latency in response to a variety of stimuli, including stress, trauma, immunosuppression and UV light [20,33]. Clinically, reactivation can result in the symptomatic recurrence of lesions in the region supplied by the portion of the trigeminal nerve implicated in reactivation [34]. Secondary lesions often occur on the vermillion border of the lip, termed herpes labialis. Unlike primary intraoral herpetic lesions, secondary herpetic lesions generally occur on the keratinized mucosa, such as the hard palate and attached gingiva [34]. While most cases of HSV-1 primary and recurrent infections are mild, serious complications can include erythema multiforme [35], encephalitis [36] and blindness [37]. Treatment of oral herpes infections is generally palliative, but viral nucleoside analogs such as acyclovir and its prodrug derivative, valacyclovir, can be used to prevent recurrent lesions if taken before active blister formation [38]. However, acyclovir resistance is a rising issue in immunocompromised individuals, who often suffer from more frequent and severe episodes of HSV-1 reactivation [38]. 

### 2.2. Epstein–Barr Virus (EBV)

Epstein–Barr virus primarily infects B cells and epithelial cells, and infection of the oral epithelium leads to several clinical manifestations in the oral cavity [39,40,41]. Infection with EBV is ubiquitous, with over 90% of the adult population affected [42]. Once a person is infected by EBV, asymptomatic shedding via saliva occurs throughout the lifetime, and viral replication in epithelial cells provides a continuous source of viral shed [7,43]. EBV infection of B-lymphocytes results in chronic latent infection, and the close association of lymphoid tissue with the oral epithelium may provide a source of EBV transfer and sustained replication in oral epithelial cells [7,44]. While primary infection with EBV during childhood is generally asymptomatic, EBV infection in young adults can lead to the development of infectious mononucleosis [41]. Throat wash samples from infected individuals have led to the identification of EBV in oropharyngeal cells [45]. Treatment for infectious mononucleosis is symptomatic, and specific antiviral therapy is not recommended [11]. EBV has also been associated with a number of epithelial and non-epithelial lesions in the oral cavity as well as oral inflammatory disease, including oral lichen planus and Sjogren’s syndrome, though a direct link is still unclear [41,46]. In addition to illness in immunocompetent hosts, EBV infection can result in further complications in immunocompromised hosts. Oral hairy leukoplakia (OHL) is a white, hyperkeratotic patch on the lateral border of the tongue common in HIV-positive individuals, and EBV lytic replication has been detected in samples from OHL lesions [47]. Importantly, EBV is also a known human oncovirus, and is linked to malignancies including nasopharyngeal carcinoma and Burkitt’s lymphoma, a non-Hodgkin’s lymphoma that is prevalent in the African continent, particularly among children [48,49]. Oral involvement is highly common in endemic Burkitt’s lymphoma, accompanied by bony expansion of the maxilla [50]. Recently, EBV co-infection with human papilloma virus (HPV) has been described in oropharyngeal carcinoma samples. This finding, along with the observation that HPV can mediate lytic reactivation of EBV in oral epithelial cells, suggests an additional role for EBV involvement in tumorigenesis in the oral cavity [51,52].

### 2.3. Kaposi’s Sarcoma-Associated Herpesvirus (KSHV)

KSHV is the most recently identified member of the human herpesvirus family, and is the etiologic agent of Kaposi’s sarcoma (KS) [53], primary effusion lymphoma [54], and multicentric Castleman’s disease [55]. KS is a neoplasm of endothelial origin and is one of the most prevalent AIDS-associated malignancies. KS lesions can be present on both cutaneous and mucosal surfaces [56]. Oral KS mainly develops on the palate, the attached gingiva, and on the dorsum of the tongue, and can be observed in up to 60% of AIDS patients [57,58,59]. Testing the survival of 138 patients with HIV-1-associated KS revealed that patients with oral KS had a 3.4-fold higher death rate than those with cutaneous KS [60]. For AIDS-associated KS lesions, combined antiretroviral therapy is currently the standard treatment [56]. KSHV is primarily transmitted orally, and viral shed in the saliva is both frequent and recurrent in infected individuals [8,24,61,62]. KSHV can infect oral epithelial cells to produce progeny virus in vitro, providing additional evidence for productive oral KSHV infection [40,63]. Additionally, studies in oral epithelial organotypic raft cultures demonstrated that epithelial differentiation induces KSHV productive infection, indicating a mechanism for viral shed from the superficial layers of the oral epithelium [64]. The abundant lymphoid tissue in the oral cavity provides a population of B cells, such as tonsillar B cells, where KSHV can establish a latent infection [65,66]. KSHV can also latently infect oral fibroblasts, which enhances the secretion of KS-promoting cytokines from infected oral fibroblasts and increases their invasiveness, highlighting the potential role for KSHV-infected oral fibroblasts in facilitating oral KS [67,68]. Moreover, the oral cavity provides a unique site of interaction between KSHV and common periodontal pathogens, such as *Porphyromonas gingivalis* and *Staphylococcus aureus*, which have been shown to be able to reactivate the virus from latency in infected oral cells [69,70,71], indicating a complex interplay between KSHV infection and the oral microbiome. 

## 3. Impact of Host Epigenetic Machinery on the Viral Life Cycle

The fundamental building block of chromatin is the nucleosome, which consists of 147 base pairs of DNA wrapped around a histone octamer, containing two copies of each of the four histone proteins, H2A, H2B, H3 and H4. The expression of genes in eukaryotes is regulated by the state of their chromatin structures, which can be either transcriptionally active (euchromatin) or repressed (heterochromatin). The regulation of the chromatin structure associated with gene regulatory DNA sequences, such as promoters and enhancers, determines the expression level of genes. Chromatin dynamics are controlled by a large variety of epigenetic factors including ATP-dependent chromatin-remodeling complexes, enzymes regulating DNA methylation and histone modifying enzymes that regulate post-translational modifications (PTMs) of histones [72,73]. Presently, more than 130 different posttranslational modifications have been identified on histones (histone marks), of which only a few have been studied so far for their role in the regulation of cellular genes and even less in the regulation of viral infections [74]. 

Viruses can hijack host chromatin-modifying enzymes to modulate their own gene expression and promote viral infection [16]. Additionally, viral-mediated dysregulation of cellular epigenetic enzymes can specifically target host genes, including those related to the DNA damage and innate immune response pathways [16]. As a result, epigenetic regulation is an important driver of tumorigenesis during oncoviral infections. While the specific mechanisms vary between oncogenic viruses, epigenetic regulation is crucial to the expression of viral oncogenes and viral deregulation of host gene expression [75]. Additionally, viruses may encode specific factors which can enhance, inhibit or redirect host chromatin modifying enzymes [16]. Since epigenetic gene regulation has been shown to be crucial for controlling many viral infections, epigenetic-targeted antiviral therapies have been increasingly explored, indicating an increased need for research into the epigenetic mechanisms that regulate viral infections [17,76]. 

**Figure 2:**
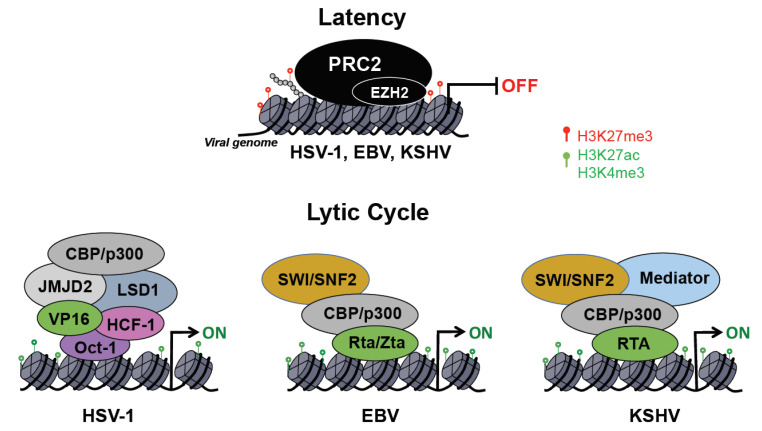
Epigenetic regulation of herpesvirus infections. During latency, lytic viral gene expression is suppressed by the polycomb repressive complex 2 (PRC2). The enzymatic subunit of PRC2 is EZH2, which deposits the repressive histone mark H3K27me3 on the viral chromatin. PRC2-mediated inhibition of lytic gene expression during latency was reported for HSV-1, EBV, and KSHV as well. During the lytic cycle, herpesvirus lytic factors (e.g., HSV-1 VP16; EBV Zta and Rta; KSHV RTA) recruit epigenetic enzymes and chromatin remodeling complexes to the viral promoters, leading to the deposition of activating histone marks (e.g., H3K27ac, H3K4me3) and chromatin changes that activate transcription of lytic viral genes. Note: the illustration of epigenetic factors does not reflect their true biological interactions in every case.

Herpesviruses have two distinct life cycles: latency and lytic. While only a few viral genes are expressed during latency, the lytic cycle is characterized by the temporally ordered induction of immediate early (IE), early (E) and late (L) lytic genes as well as viral DNA replication and virus production. Upon entry into the host cell nucleus, the herpesvirus DNA genome undergoes circularization and chromatinization within the first hours of infection [77]. One of the hallmarks of the herpesviruses is the ability to establish chronic infections, which is accomplished by the establishment of latency. In this dormant state, the viral DNA has a stable chromatin structure, similar to that of the cellular genome, and persists in the nucleus of infected cells as a non-integrated, circular mini-chromosome (episome), while the lytic gene expression is repressed through various chromatin and DNA modifications [78,79]. Diverse external and internal stimuli can evoke herpesvirus reactivation from latency, leading to chromatin changes on the viral episome, which promote the de-repression and full expression of lytic viral genes [78]. Here, we specifically focus on the epigenetic regulation of HSV-1, EBV, and KSHV during infection of oral epithelial cells, which results in detectable virus load in the oral cavity of infected individuals (Figure 2).

### 3.1. Transcriptional and Epigenetic Control of HSV-1 Infection

Following the initial lytic replication in epithelial cells, HSV-1 establishes latent infection in sensory neurons. During lytic infection the viral DNA is associated with histones enriched with activating histone marks such as H3K4me3 and histone acetylation that support lytic viral gene expression [80,81]. Lytic viral factors are also crucial to circumvent host epigenetic repression of lytic viral genes to promote viral replication. The VP16 viral protein is part of the HSV-1 virion and is therefore delivered into cells during infection. Importantly, VP16 interacts with Oct-1 and host cell factor 1 (HCF-1), forming a transcriptional regulatory complex, which is essential for inducing lytic viral genes following primary infection of epithelial cells [82,83]. Nuclear localization of the VP16 and HCF-1 complex in epithelial cells is a critical step in initiating the lytic gene cascade, whereas the cytoplasmic localization of these factors in neuronal cells can promote viral latency [79,84,85]. In the nucleus, VP16/HCF-1 associates with octamer binding transcription factor (Oct-1) to form enhancer core complexes at viral immediate early (IE) genes. HCF-1 in turn recruits several histone-modifying complexes, including H3K9 demethylases (e.g., LSD1, JMJD2), H3K4 methyltransferases (e.g., Set1/MLL) as well as histone acetyltransferases (e.g., CBP/p300) (Figure 2) [79,86]. This step in the HSV-1 productive infection is a potential target for epigenetic-directed therapy, as it has been shown that inhibition of H3K9 demethylase LSD1 or JMJD2 can inhibit lytic viral gene expression and reactivation from latency [87,88].

The accumulation of activating histone marks on the viral genome during initial HSV-1 infection leads to the expression of HSV-1 immediate early (IE) genes, including ICP0 and ICP4, which further enhance the expression of other lytic genes. ICP0 disrupts the transcriptionally repressive CoREST histone deacetylase and ND10 complexes, leading to a euchromatin state at early and late gene promoters and allowing expression of the entire cascade of lytic viral genes [89,90,91]. ICP4 also contributes to the activation of viral transcription by preventing the formation of silencing nucleosomes on HSV-1 genomes [92]. The host innate immune response to viral infection includes many factors that sense foreign virus elements to initiate a downstream antiviral response. One such host factor that has been characterized in HSV-1 infection is interferon-inducible factor IFI16, which recognizes viral DNA and facilitates the recruitment of repressive chromatin modifications to the HSV-1 genome [93,94]. During de novo HSV-1 infection of oral keratinocytes, IFI16 is degraded in an ICP0-dependent manner, demonstrating a viral mechanism to maintain an active chromatin state in the oral cavity [95,96]. 

After lytic replication in the oral mucosa, HSV-1 virions infect cells of the sensory ganglia in the trigeminal nerve, where a chronic latent infection is established. It is now understood that chromatin state determines HSV-1 latent versus lytic states, wherein heterochromatin formation in sensory neurons limits lytic viral gene expression [84]. In neuronal cells, all regions of the HSV-1 genome with the exception of the latency-associated transcript (LAT) are associated with repressive chromatin marks. For example, the polycomb repressive complex 2 (PRC2) histone methyltransferase EZH2 deposits H3K27me3 histone mark in lytic gene regions to limit gene expression in latently infected neurons [97]. Bmi1, a member of the polycomb repressive complex 1 (PRC1) has also been demonstrated to bind to specific sites in the HSV-1 genome, which can contribute to viral gene silencing during latency. Interestingly, treatment of human foreskin fibroblasts with small molecule inhibitors of EZH1/2 induced a cellular antiviral immune response, thereby suppressing HSV-1 lytic infection, indicating another possible epigenetic drug target for herpesvirus infections [98]. Further details about the regulation of viral latency and reactivation in neurons both in vitro and in mouse and rabbit models can be found in recent publications [99,100,101,102].

### 3.2. Epigenetic and Transcriptional Principles Governing EBV Infection

EBV infection of oral epithelial cells provides a primary source of viral shed, and as a member of the gammaherpesvirus family, EBV then establishes a chronic infection of B cells [7]. While we know little about how EBV DNA acquires chromatin and its different epigenetic modifications following de novo infection, we know much more about the chromatin state of EBV DNA in infected B cells. During latent infection, several copies of the EBV episome are maintained in the host nucleus with lytic gene expression repressed by multiple different mechanisms [103,104,105]. EBV demonstrates four distinct levels of latency, classified as latency 0-III, in which promoter switching dictates latent gene expression, including subsets of the six Epstein–Barr nuclear antigens (EBNA) and three latency membrane proteins (LMP) [106]. Diversity in repressive versus active chromatin mark deposition on individual classes of latency genes contributes to differential gene expression during respective latency stages, with genome-wide correlation between repressive H3K9me3 marks and DNA methylation [107]. 

During latency, actively transcribed latent gene promoters are enriched in the activating H3K4me3 histone mark, while repressive H3K9me3 and H3K27me3 marks limit the expression of lytic viral genes [103,108,109,110]. Host factor CCCTC-binding factor (CTCF) insulates segments from the EBV genome to separate repressive and activating histone modifications to regulate the various stages of EBV latency [108,111]. De novo CpG methylation by host DNA methyltransferases is a slower process, with gradual increases in DNA methylation marks in the weeks following initial infection, suggesting that DNA methylation plays a role in stabilizing the latent genome [112]. Latent viral proteins can also modify host chromatin, such as in the case of latent factors EBNA3A- and EBNA3C-dependent recruitment of PRC2 to deposit repressive H3K27me3 marks on host tumor suppressor genes [107,113]. In nasopharyngeal carcinoma cells, EBV infection has also been shown to be associated with loss of H3K4me3/H3K27me3 bivalency in the promoter regions of crucial DNA damage response genes, demonstrating a viral mechanism of host chromatin regulation that may drive tumorigenesis in the epithelium [114]. 

B cell activation triggers EBV reactivation from latency, whereby the viral switch protein Zta is expressed along with immediate-early protein Rta, which together drive the ordered expression of the EBV lytic gene cascade [115]. Zta acts as a pioneer factor, binding preferentially to methylated viral promoters co-localizing with repressive H3K9me3 marks to recruit chromatin remodelers [110,116,117]. Interestingly, DNA methylation is required for Zta-driven lytic gene expression, and EBV must first establish a latent infection before it is able to complete its lytic cycle [118]. During reactivation, PRC2 is released from the viral genome, leading to a decrease in H3K27me3 marks [117]. Zta also recruits CBP acetyltransferase to deposit activating histone marks, and this Zta-dependent release from epigenetic repression allows for the progression of the viral lytic cycle (Figure 2) [119]. 

While EBV latency programs have been widely characterized in the context of B-cell infection, EBV is generally thought to establish a lytic infection in the oral epithelium, with lytic gene expression detected in tonsillar epithelium and oral hairy leukoplakia epithelial lesions [120,121,122,123,124]. However, latent EBV gene expression has also been detected in oral epithelial dysplasia and oral squamous cell carcinoma samples, with an enhanced expression of latent genes in more dysplastic tissue [123,125]. To study latent epithelial infection that may drive carcinogenesis, a human telomerase-immortalized normal oral keratinocyte (NOK) cell line has been developed as a model of latent EBV infection [126]. Transient infection of NOKs results in a shift in host CpG methylation patterns and a resultant modulation in host cell gene expression, with impaired differentiation ability of the oral keratinocytes upon external differentiation stimuli [127]. In NOK cells, many lytic gene promoters exist in an unmethylated state, and therefore EBV Rta, rather than EBV Zta, is necessary to reactivate these cells from latency, indicating a divergent mechanism of latent gene regulation in an oral cell line [128]. Moreover, specific differentiation-dependent cellular transcription factors in the oral keratinocytes are key to expression of Zta and Rta factors and induction of the lytic cycle [129,130,131]. Enhanced cell motility and invasiveness upon transient EBV infection of NOKs is maintained after loss of the virus, indicating the ability of viral infection to confer lasting epigenetic changes in oral cells [132].

### 3.3. Epigenetic Regulation of the Biphasic Life Cycle of KSHV

There is no well-established animal model to study KSHV infection in vivo, although oral infection of common marmoset was shown to be able to recapitulate some aspects of KS development in a non-human primate model [133]. Thus, the vast majority of studies on the regulation of KSHV infection and viral transmission rely on studying infection of various cell culture models. In contrast to HSV-1, the default pathway for KSHV infection is the establishment of latency in most cell types, while oral epithelial cells and dermal lymphatic microvascular endothelial cells have been demonstrated to support lytic primary KSHV infection to some degree [63,134,135,136,137]. Importantly, the expression of IE gene ORF50 encoding the replication and transcription activator protein RTA is necessary and sufficient to induce the lytic cycle of KSHV both in latently infected cells and following de novo infection [138,139]. RTA can induce lytic genes by binding to their promoters and recruiting epigenetic factors such as CBP/p300, SWI/SNF, mediator, and histone demethylases that can alter the viral chromatin to favor viral gene expression (Figure 2) [140,141,142,143]. Since RTA has an E3 ubiquitin ligase activity, it can also promote lytic gene expression by inducing the degradation of host proteins repressing KSHV gene transcription [144,145,146].

Most of the information on the epigenetic regulation of the KSHV genome comes from studies using latently infected primary effusion B cell lymphoma (PEL) cell lines that were originally isolated from KSHV^+^ patients. In latently infected PEL cells, the latency-associated locus of the KSHV genome is enriched only in activating histone marks (AcH3 and H3K4me3), whereas there is a mutually exclusive distribution of both activating (AcH3 and H3K4me3) and repressive (H3K9me3 and H3K27me3) histone marks throughout the rest of KSHV genome [147,148]. EZH2, the histone methyltransferase subunit of PRC2, is responsible for the global deposition of repressive H3K27me3 marks on the viral genome, and shRNA depletion or chemical inhibition of EZH2 can lead to the upregulation of lytic genes [148]. Importantly, the histone mark patterns and many of the other epigenetic regulations of the latent KSHV genome in PEL cells have been confirmed in a number of different KSHV-infected adherent cell types and in Kaposi’s sarcoma tissues as well [139,147,149].

Strikingly, prior to the establishment of KSHV latency, a number of lytic viral genes that possess immunomodulatory and antiapoptotic functions were shown to be transiently expressed following de novo infection [150]. This burst of lytic gene expression can be explained by the viral genome’s association with euchromatin in the first 24 h of primary infection and lacking of the PRC2-regulated heterochromatin [139]. However, by 72 h post-infection, the enrichment of activating histone marks such as H3K27ac and H3K4me3 declines and they are restricted to specific genomic regions, while the binding of PRC1 and PRC2 factors and their corresponding histone marks H2AK119ub and H3K27me3 increase genome-wide on the KSHV genome [139,151,152]. These chromatin changes are accompanied by the suppression of lytic gene transcription while latent genes are continuously expressed. The observed temporal and spatial distribution of histone marks along the viral genome during establishment and maintenance of latency have been linked to the controlled recruitment of histone modifying enzymes to the viral episomes [139,152,153]. The mechanism of the specific spatial and temporal targeting of epigenetic factors to the KSHV genome is still largely unknown. However, in some cases, specific KSHV proteins, the viral long non-coding PAN RNA, and the unmethylated CpG motifs in the viral DNA have been implicated in the regulation of the recruitment of host epigenetic factors to the viral episome [152,154,155]. A recent siRNA screen of host epigenetic factors during de novo KSHV infection identified several new players in the formation of the KSHV epigenome and establishment of latency beyond PRC1 and PRC2, which includes the histone demethlyase KDM2B, NuRD and Tip60 repressive complexes among many others [156]. It is likely that the binding of all of these host epigenetic factors contribute to the different epigenetic layers of the KSHV episome, which can support the establishment and maintenance of the chromatin structure of latent viral episomes.

KSHV lytic gene expression and replication in oral epithelial cells after primary infection raises the question of how these cells are different from other cell types that do not support de novo lytic infection. Investigation of KSHV-infected immortalized gingival epithelial cells revealed that while there was an increased amount of H3K4me3 and H3K27ac on both latent and lytic viral promoters, deposition of the repressive H3K27me3 and H2AK119ub histone marks was very low [139]. Interestingly, lower expression of polycomb group proteins was detected in oral epithelial cells supporting KSHV lytic infection than in cells that did not support lytic viral infection, indicating a potential rationale for the ability of the virus to establish productive infection in the oral cavity [139].

Moreover, a recent siRNA epigenetic screen identified histone demethylase KDM2B as a key restriction factor of lytic gene transcription not only in latent infection but also in the lytic infection of primary gingival epithelial cells [156]. KDM2B is a histone demethylase associated with the removal of activating H3K4me3, H3K36me2 and H3K79me2 marks, and has also been shown to play a role in PRC1 recruitment to specific regions in the host genome [157,158,159,160]. The viral gene expression inhibitory function of KDM2B was linked to its histone demethylase and DNA-binding activity [156]. KDM2B protein levels can be dysregulated by different environmental stimuli such as hypoxia, which increases KDM2B expression, and this can affect the outcome of KSHV infection [161,162]. Interestingly, Naik et al. recently showed that while KDM2B normally acts as a suppressor of KSHV lytic gene expression, overexpressed KDM2B promotes lytic gene expression during de novo KSHV infection through interaction with the E3 ubiquitin ligase SCF complex [162]. It was demonstrated that the SCF^KDM2B^ complex increases the half-life of c-Jun protein during KSHV infection leading to elevated AP-1 activity, which supports lytic viral gene expression [162]. Collectively, these studies indicate that the same host epigenetic factor can both inhibit and promote lytic KSHV infection in a context dependent manner by either directly altering the viral chromatin or modulating host signaling pathways that affect viral gene expression. In fact, several histone-modifying enzymes have been described to regulate biological processes other than chromatin-based gene regulation [163,164,165,166,167]. Thus, the effects of host epigenetic enzymes on KSHV infection have to be interpreted cautiously and in the context of the circumstances of viral infection.

Both the expression and activity of histone modifying enzymes can be influenced by various environmental stimuli, which can affect viral pathogenesis. Recent studies have shown that the oral microbiome can leave its imprint on oral cells through secreting metabolic byproducts which dysregulates host epigenetic factors. It was demonstrated that short-chain fatty acids produced by periodontal pathogens (e.g., *P. gingivalis*, *F. nucleatum*) can either inhibit the enzymatic activity or downregulate the expression of host epigenetic factors (e.g., EZH2) that repress KSHV gene expression thereby promoting KSHV replication in the oral cavity [71,168]. Moreover, exosomes from saliva of HIV^+^ individuals have been shown to enhance lytic KSHV infection of human oral epithelial cells. This effect was linked to HIV TAR RNA enriched in the exosomes, but the mechanism of how the viral TAR RNA induces KSHV gene expression remains unresolved [169]. Nevertheless, these studies indicate that KSHV replication can benefit from both bacterial and other viral infections in the oral cavity, which can increase shedding of KSHV into saliva and KSHV transmission.

## 4. Outlook for Epigenetic-Directed Therapeutic Interventions for Viral Infections

Oral transmission and infection of oral epithelial cells is conserved among many viruses, and a range of oral pathologies associated with viral infections include but are not limited to the herpesvirus family [6,11,13,29,170]. Epigenetic drugs have gained increasing interest in the field of cancer research, and many of these drugs have been approved for the treatment of select cancers [171]. Given the significance of epigenetic regulation of oral viral infection, many of the cancer epigenetic drugs can also provide a promising avenue for therapeutic intervention of viral infections [17]. HIV has served as the hallmark for the study of epigenetic manipulation in antiviral therapeutics. Two paradigms, deemed the “shock and kill” and “block and lock” strategies, have been developed. In the “shock and kill” paradigm, HIV-infected cells are treated with histone deacetylase, DNA methyltransferase and/or histone methyltransferase inhibitors to induce reactivation of the virus from latency, which makes the infected cells and the virus visible for the immune system to eliminate the latent viral pools [17,172,173]. Alternatively, the “block and lock” paradigm aims to push latent HIV reservoirs into tight latency through inhibition of Tat-dependent transcription [17,174]. Epigenetic drugs have also been tested in the context of other chronic viral infections, such as in the use of bromodomain and extra-terminal domain (BET) inhibitors to target HPV and herpesvirus infections [175,176]. Similarly, inhibitors of lysine-specific histone demethylase 1 (LSD1) and PRC2 enzymatic subunit EZH2 have been shown to blunt HSV-1 reactivation [98,177,178]. However, the specific in vivo applications of epigenetic drugs in the context of oral viral infection have yet to be explored. 

## 5. Conclusions

The impact of epigenetic regulation on viral infection has been a field of increasing interest, leading to an enhanced understanding of the interplay between key virus and host factors in the progression of the viral life cycle. The reversibility of epigenetic modifications creates an opportunity to use epigenetic-targeted therapies to both treat and prevent viral infections. The oral cavity plays a crucial role as a viral entry site and as a site that is permissive to productive herpesvirus infection. As a unique site of host–virus interaction, the epidemiologic relevance of oral transmission as a major source of viral spread warrants further study into the regulation of the viral life cycle in the oral cavity.
